# Process evaluation of a pre-adolescent transdisciplinary health intervention for inter-generational outcomes

**DOI:** 10.1371/journal.pone.0261632

**Published:** 2021-12-23

**Authors:** Keshni Arthur, Nicola Christofides, Gill Nelson

**Affiliations:** 1 School of Public Health, Faculty of Health Sciences, University of the Witwatersrand, Johannesburg, South Africa; 2 UCL Institute for Global Health, University College London, London, United Kingdom; University of Pretoria, SOUTH AFRICA

## Abstract

**Background:**

*The CIrCLE of Life Initiative* was implemented to 537 grade 6 learners and their parents, in five Government-run South African primary schools. The transdisciplinary intervention was intended to increase knowledge and skills on HIV and obesity. The study aim was to assess and report on the implementation process.

**Methods:**

Data was collected on an adapted Proctor’s taxonomy of implementation outcomes, and to assess participants’ experiences. Qualitative and quantitative data were collected through educator logbooks, researcher observations, and learner-parent workbooks.

**Results:**

Differentiations between the various school contexts were observed. The process evaluation revealed high learner penetration (97.2%), but lower learner and parent exposure (44.3% and 55.5%, respectively). All educators thought that the intervention was a fit for both rural and urban schools, different socio-economic groups, and people of different ethnic and cultural backgrounds. The intervention was perceived to be sustainable, and there were recommendations for adoption into the school curriculum and scale-up if found to be effective.

**Conclusion:**

The process evaluation facilitated the assessment of the implementation outcomes, described its processes, and acknowledged fundamental characteristics that could justify variability in the intervention impact and outcomes. The value of process evaluations and their benefit to the science of implementation were demonstrated.

## Introduction

South Africa, like many other developing countries, is in the midst of a health transition that is characterised by the double burden of communicable disease (CD) and non-communicable disease (NCD). While the country continues to deal with the challenge of CDs, such as human immunodeficiency virus (HIV), acquired immune deficiency syndrome (AIDS), and tuberculosis (TB) [1], the upsurge in obesity [2] and accompanying NCDs [3], such as cardiovascular disease and diabetes, present a novel set of problems.

The double burden of disease is a public health concern that creates a complex environment for intervention delivery, particularly for diseases such as HIV [4] and emerging threats such as obesity [5]. Both HIV and obesity are areas of complex intervention research and often include multiple related components, target various ages of the population, and have a wide range of interconnected outcomes [4, 6–8]. While there have been several studies aimed at HIV and obesity prevention, a limitation to the interpretation of the study outcomes is the poor reporting of process measures in such studies [9]. A recent Cochrane review of childhood obesity prevention studies [10] found that of 39 trials targeting children, around a third did not report process measures. The authors recommended that future studies need to report comprehensive process evaluation data in order to interpret outcomes and to guide future implementation [11].

Evidence from other reviews have identified the contribution of health promotion programmes in schools and its benefits in improving the health of children [12, 13]. However, an understanding of how the fundamentals of such interventions can be best implemented remains overlooked [14]. The reality of implementing programmes in a school environment involves the adaptation of programmes to be a fit with the local context [15] within a broader educational and health system and the committed engagement of stakeholders [16, 17]. The implementation of such programmes is frequently dependent on the behaviour of stakeholders (participants and implementers) within the system [18]. An understanding of how the effects of such programmes are attained is less common, but essential approach [19].

With the limited success of conventional approaches in addressing the double burden of CD and NCD, health interventions using transdisciplinary approaches to prevent such diseases could provide holistic healthcare solutions across disciplinary boundaries [20]. Transdisciplinary interventions comprise individuals from different disciplines working jointly to implement conceptual, theoretical, methodological, and translational programmes that integrate and move beyond discipline-specific areas to address a common problem that could provide holistic, collaborative solutions across disciplinary boundaries [20, 21]. The engagement of the education sector as a participant in an integrated transdisciplinary health promotion and prevention intervention offers an ideal strategy to target the complexities of HIV and obesity. *The CIrCLE (Child Influencing paRent Communication for Life Education) of Life Initiative* was developed as an intervention to target pre-adolescent learners and their parents for short- and long-term benefits [22].

Implementation science methodology aims to close a gap between research and practice [23] by identifying and evaluating the processes necessary to ensure successful widespread translation of evidence-based practices (EBP) into real-world settings [24]. Intervention evaluation studies often focus on their content and immediate effects, with less emphasis on the processes that may explain, sustain or improve these effects. A criticism of outcome evaluations is the difficulty in explaining the processes by which the results of the interventions were attained [25].

Implementation of *The CIrCLE of Life Initiative*, therefore, underwent both an outcome (intervention effectiveness) and process (implementation effectiveness) evaluation. Intervention effectiveness is a necessary, but not sufficient, condition for achieving positive intervention outcomes [26]. Intervention effectiveness is differentiated from implementation effectiveness and is a critical and effective approach to delineating between intervention and implementation failure. When implementation efforts of an intervention in a new setting fail, it is important to know if the failure occurred because the intervention was ineffective in the new setting (intervention failure) or if it was deployed incorrectly or weakly (implementation failure) [27]. Only through high-quality implementation can anticipated benefits and intervention effectiveness be achieved. Implementation outcomes serve as necessary preconditions for attaining subsequent desired changes in clinical or service outcomes because an intervention will not be effective if it is not implemented well [25].

For a comprehensive evaluation effort emphasis has also been placed on evaluating intervention processes [28]. Process evaluations decipher inconsistencies between expected and observed outcomes and inform future implementation efforts. An essential role for process evaluations is to examine the quantity and quality of what was implemented [29]. A process evaluation may be used to monitor and report fidelity of intervention implementation (i.e. whether the intervention was delivered as intended) [30, 31], quality of implementation [32], clarify causal mechanisms and identify contextual factors [25] associated with variation in intervention delivery and outcomes. Reliable, valid, and pragmatic evaluation is necessary for monitoring and assessing the success of implementation efforts and comparing the effectiveness of alternative implementation strategies. Studies have shown the value and tools to be used in a comprehensive process evaluation [33, 34].

The study aims to assess and report the results of the process evaluation of the implementation of *The CIrCLE of Life Initiative*. The process evaluation will be used to justify any variability in intervention impact and outcomes.

## Methods

The process evaluation was conducted alongside a pretest posttest outcome evaluation of *The CIrCLE of Life Initiative*. Although both evaluations were done concurrently, the results were analysed separately and reported as such. All process evaluation data were analysed before the outcome evaluation results were available to minimise the risk of bias in interpretation [18].

S1 File presents a Standards for Reporting Implementation Studies (StaRI) checklist with details regarding the implementation strategy that was used during the study [35].

Ethical approval was obtained from the Human Research Ethics Committee of the Health Sciences Faculty, University of the Witwatersrand (clearance certificate no. M180220). The Gauteng Department of Education granted permission to conduct the intervention at schools. Written informed consent was obtained from all participating educators. Written informed consent and assent to participate in the study were also obtained from the participating parents and learners, respectively.

### The conceptual framework for implementation outcomes

Five outcomes from the taxonomy of the eight conceptually distinct implementation outcomes (acceptability, adoption, appropriateness, cost, feasibility, fidelity, penetration, and sustainability) proposed by Proctor et al. (2011) [27] formed the basis of the process evaluation. Adoption, cost and feasibility outcomes are not reported in this paper. Acceptability is defined as the perception among implementation stakeholders that a given service, practice, or intervention is satisfactory. Appropriateness is the perceived fit, relevance or compatibility of the intervention or evidence-based practice for a given practice setting, provider or consumer. Penetration is defined as the integration of a practice within a service setting and its sub-systems. Sustainability is the integration of a given program within an organisation’s culture through policies and practices [27].

Fidelity is repeatedly highlighted as a crucial component of process evaluation efforts in various process evaluation frameworks [27, 36–38]. Dane and Schneider (1998) [38] further define five dimensions of fidelity: adherence (how well the intervention delivery followed recommended methods), exposure (the amount of intervention received by the participants), quality of delivery of the intervention, participant responsiveness (how the participants responded to the different intervention components) and programme differentiation (identifying whether certain aspects of the intervention were more effective than others) [38].

Although not part of the Proctor et al. [27] framework, context (environmental aspects of the intervention setting) is an additional dimension in this report as it has been acknowledged as a key dimension by Linnan and Steckler (2002) [36].

### Overview of the intervention

**Fig 1:**
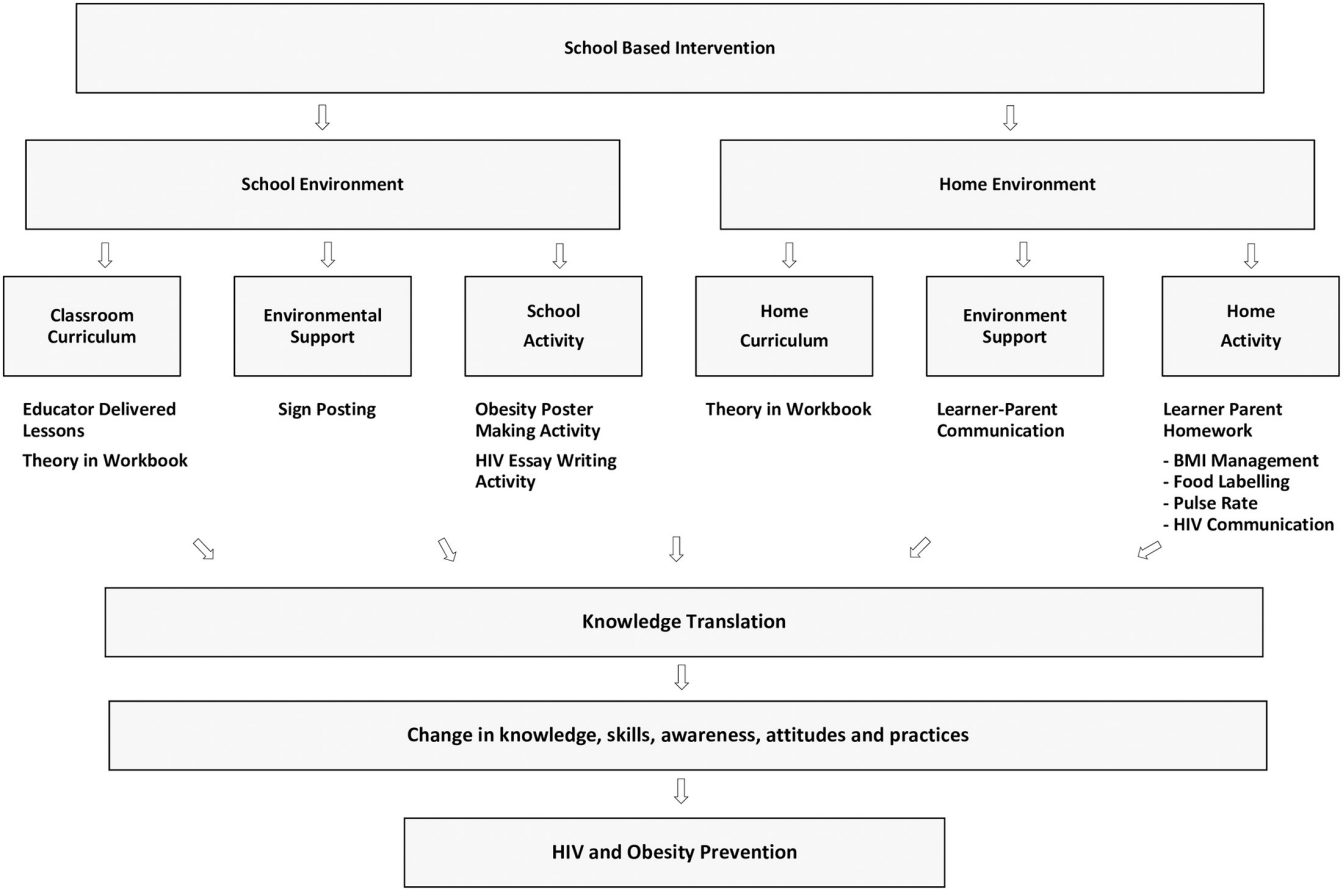
The CIrCLE of Life Initiative. Reprinted from Arthur et al. (2020) under a CC BY license, with permission from Health Education Journal, original copyright 2020 [22].

Full details of the intervention design and programme are described elsewhere [22]. In summary, *The CIrCLE of Life Initiative* (Fig 1) had both a school and home component, each of which comprised a learning curriculum, environmental support, and activity-based constituents that aimed to increase learner knowledge and skills and to engage caregivers. Trained educators delivered lessons about HIV and obesity to all Grade 6 learners at the school. The classroom curriculum for learners required delivery of a five-hour face-to-face intervention delivered weekly through 10 30-minute lessons. Learners were asked to communicate their learnings to their parents/caregivers at home. Parents/caregivers were requested to read through the lesson in a workbook and sign in acknowledgement of reading. The workbook contained shared learner-parent homework activities that would take approximately half an hour per week. The intervention was carried out within one school term equivalent to three months.

Educators were provided with training and resources. Each educator was given a training manual and trained on the implementation strategy and content (Table 1). Most educators understood the content as the content was similar to what they were accustomed to teaching due to the inclusion of such topics in the national school curriculum. However, the teaching methods and the workbooks were novel. *The CIrCLE of Life* curriculum was designed to fit in with the national school curriculum, and certain aspects overlapped as depicted in Table 1. Although familiar with some skills that were already in the syllabus, such as food label interpretation, additional time was spent supporting educators with skills such as measuring Body Mass Index (BMI) and pulse rate. The communication aspects and the shared homework activities were new approaches.

**Table 1:** Overview of lesson content.

	Content	Skills	Reinforcing Activity	Parent-Learner Homework
**Lesson One**: Obesity	Healthy body image *	How to measure weight	Diary of a healthy kid	Measure and Interpret Learner’s BMI
	Underweight, Overweight and Obesity	How to measure height	Crossword puzzle	
	Body Mass Index (BMI)	Interpreting BMI		
**Lesson Two**: Nutrition	Healthy eating *	How to interpret a food label *	Word search puzzle	Interpret a food label at home
	Food-based dietary guidelines *		Colouring-in-picture	
**Lesson Three**: Physical Activity	Benefits of physical activity	Measuring and interpreting pulse	Colouring-in-picture	Measure and Interpret family member’s pulse
	Heart rate (pulse)			
				Obesity poster activity
**Lesson Four**: HIV/AIDS	HIV/AIDS *	Reducing the risk of HIV/AIDS	Crossword puzzle	HIV/AIDS story-writing activity
	Understanding the risks of HIV/AIDS			
**Lesson Five**: Communicating about HIV/AIDS	Communication	Dealing with discomfort and support for sexual-specific communication	Communication game	Family communication exercise
	Importance of communication			
	Healthy ways of communicating			

* Already part of the existing Grade 6 national school curriculum

Reprinted from Arthur et al. (2020) under a CC BY license, with permission from Health Education Journal, original copyright 2020 [22].

Although the lessons were designed as part of the life skills lesson, the implementers could choose to deliver them during other subject lessons—as part of a transdisciplinary lesson. For example, measuring BMI could be covered during the mathematics lesson to reinforce the calculations, and pulse rate could be taught during the physical education lesson while exercising.

### Setting

In South Africa, schools (urban and rural) are categorised into five groups or quintiles [39], based on the relative wealth of their surrounding communities. Schools in the most impoverished communities are classified as quintile 1 and schools serving the wealthiest communities, as quintile 5. Quintiles 1, 2 and 3 schools do not charge fees. The Government subsidises schools accordingly; quintile 1 schools receive the highest allocation per learner and quintile 5 schools receive the lowest [39].

Five government-run primary schools in a district located in Gauteng province, South Africa were purposely selected to represent each of the quintiles and invited to participate. Two schools were classified as quintile 1, and three as quintile 2, 3 and 4. The quintile 4 school was the only fee-paying school and the only school that did not provide a feeding scheme. Three of the schools were in a rural area while two were in an urban area. The sizes of the schools range from 650–1100 learners with a teaching complement of 25–30 educators. There are approximately 100 Grade six learners in each school.

### Participants

Seven educators were recruited and agreed to implement the program in 12 Grade 6 classes. A total of 537 Grade 6 learners and their parents/caregivers were invited to participate. All learners and their caregivers were exposed to the intervention activities, and the process evaluation, therefore, included all learners and parents.

### Data collection

A mixed-methods approach [40] was employed; quantitative and qualitative data were collected concurrently between July and September 2018.

A pragmatic approach to data collection was adopted to maximise response while minimising the impact on intervention delivery and the workload for educators. Data collection methods and tools included: 1) checklists and logbooks completed by intervention providers (educators); 2) interviews with educators; 3) observations of lesson delivery and context; and 4) use of administrative data, such as attendance registers. Tools were selected to ensure that the resulting data would address the defined evaluation dimensions. Multiple methods were used to measure the same evaluation dimension in order to triangulate findings and to ensure that data were available for each dimension.

Logbooks were designed to collect self-reported data about intervention delivery from educators. The logbooks collected information on acceptability, appropriateness, penetration, sustainability, and the fidelity dimensions: adherence; exposure; participant responsiveness; and programme differentiation. The logbooks documented both quantitative (e.g. how many learners attended the lesson) and qualitative data (e.g. details of how the lesson was delivered, problems experienced). Processes were assessed by the completion of an implementation checklist in the logbook. The tool allowed for dichotomous (yes/no) and open-ended responses.

Data from learner-parent workbooks were extracted to document both quantitative data (e.g. how many activities were completed/signed by parents). The results are based on 388 learners who handed in their workbooks at the end of implementation of the intervention. Based on daily observations and the number of stickers that had been handed out, more than 388 learners had completed their homework and attained their parents’ signatures, but the balance did not hand in their workbooks. Most of the workbooks not submitted were from the quintile 1, 2 and 3 schools; only three were from the quintile 4 school.

A single researcher undertook direct observations of intervention delivery at every class. Fieldnotes of implementation experiences were documented. Behavioural observations or field notes were used to provide an assessment of intervention implementation. The quality of delivery (i.e. educator–learner interactivity, educator competence of employing teaching methods, processes, teacher enthusiasm, learning principles, learner engagement, and learner attentiveness) was noted through observations, to determine how well lessons were delivered and received. A comprehensive observation tool was used to document the observations and included a checklist and qualitative descriptions of the implementation of the intervention. All data were captured with the informed consent from the educators.

Data were collected for each intervention component and all implementation outcomes. A reliability cross-check was performed between all data sources to ensure accuracy of reporting. Triangulation of the data was necessary to create an accurate and holistic picture of the intervention implementation and the associated response.

### Data analysis

All data were captured using RedCap. The quantitative data were exported into Microsoft Excel, where ratios were calculated to show learner and parent, penetration and exposure rates. Learner penetration was calculated as the ratio of the number of learners who attended a given lesson or participated in an activity to the total number of learners in the grade. Learner exposure was the ratio of the number of learners who completed the homework activities to the total number of learners in the grade. Parent exposure was the ratio of the number of parents who signed the workbook to the total number of learners in the grade. Dose delivered (what the programme delivered) was the function of the efforts of the educators and was calculated by the number of intended lessons of each intervention component delivered.

Qualitative data collected from the educator logbooks and transcribed interviews with educators were analysed. Content analysis [41] was conducted, where all data were systematically transformed into an organised and concise summary of key results. The qualitative data were used to better understand how each intervention component was delivered and its influence on various factors during delivery.

## Results

The descriptive information about participating schools, classes and educators are shown in Table 2. The highest educator-learner ratio per grade at schools was a quintile 1, rural school with 54 learners to one educator; the smallest was another quintile 1, rural school with 39 learners to one educator.

**Table 2:** Descriptive information about participating schools, classes and educators.

	Quintile 1 Rural school 1	Quintile 1 Rural school 2	Quintile 2 Rural school	Quintile 3 Urban school	Quintile 4 Urban school
	(n)	(n)	(n)	(n)	(n)
**Grade 6 life skills educators per school**	2	1	1	2	1
**Grade 6 classes per school**	3	2	2	3	2
**Grade 6 learners per school**	116	107	79	142	93
**Educator-learner ratio per grade**	39	54	40	47	47
**Lessons delivered per school**	30	20	20	30	20

There was 96.7% adherence to lesson delivery where all components were delivered by educators, at each school. The dose of lessons delivered in each class, at every school was 10 lessons. Lessons were delivered during a 30 minute period. The frequency of lessons was twice a week over one school term. One educator missed the delivery of four of her lessons, due to absenteeism without leave and the researcher stepped in at the request of the head of school to deliver the sessions.

### Context of implementation

Differences were observed between the various contexts of implementation. At the quintile 3 school, the class sizes ranged from 45–50 learners. As a result, classrooms were packed and noisy. Learners at this school shared the small desks, and some sat on buckets instead of chairs. The other four schools had larger classrooms that were well-ventilated. Learners from four of the five schools had to carry around their chairs to different classes for various lessons. The classrooms at these four schools were also very dusty. A train track was located adjacent to the quintile 2 school, and the learners were distracted whenever a train passed. Classes at this school were always rowdy, and most learners did not pay attention or engage in the lesson.

The headmasters from both quintile 1 schools stated that many of their learners were affected by HIV and its consequences. Many of their learners came from orphanages or lived with guardians, e.g. grandparents. High-risk behaviour was also common at these schools. “*Drugs*, *smoking and sex is rife here*. *Many of the girls are being raped by elders but don’t even know what it is*” (Quintile 1, rural educator).

Educators from non-fee paying schools (quintiles 1, 2, 3) also complained that parents had no interest in their children’s school work, and did not attend progress meetings or collect reports from schools (it is compulsory for parents to collect reports).

The availability and provision of food varied by school. Non-fee paying schools had funded feeding schemes for the learners, while learners from the quintile 4 school brought their own lunch.

The school physical activity environments also differed. Only two schools (a quintile 1 and the quintile 4) had grassed fields. The other three schools had dusty areas, with hard soil as fields/playgrounds. Only the quintile 4 school provided sporting facilities and equipment.

Other than the quintile 4 school, all school management teams were welcoming and excited that their school had been chosen as a study site. The quintile 4 school’s management acted as ‘gatekeepers.’ They often cancelled appointments and limited the researcher’s interaction with the learners. Learners were also given inaccurate details about the programme which discouraged participation. However, following talks with the life skills educator, the implementation was supported and the initial negative interactions with the school improved to more favourable interactions.

### Fidelity and valence of adaptations

The process evaluation revealed strong implementation fidelity and high dose delivered. Observations and logbook analysis showed that there was no programme adaptation of any critical components. Adherence indicators were based on the delivery of the programme content, methods and activities (Table 1). All schools adhered to the intervention protocol and lessons were delivered according to plan, although there were a few positive adaptations.

One educator who taught two classes at the quintile 3 school reinforced the lesson content by translating it into Setswana and isiXhosa. The learners in the two classes were split according to these home languages. When she did this, many learners stopped what they were doing and give her their undivided attention and nodded their heads in acceptance. This communication technique and understanding was not witnessed at any of the other classes at this school or in the other four schools.

Another adaptation, recommended in the manual, was to conduct one of the lessons that incorporated exercise in the open field, instead of the classroom. A quintile 4 educator did this.

Another manual-recommended adaptation made by the quintile 3 school was to conduct the story-writing component during the English lesson. In preparation for one of the lessons, another quintile 3 educator taught the ‘Macarena’ dance to the learners. During the lesson which was conducted in the life skills lesson, she continuously made inferences to the dance and the song.

At the quintile 3 and 4 schools, one of the lessons required additional time to complete and extended into the break. “*Taking measurements in Lesson 1 became a challenge as more time was needed*, *especially when taking both weight and height measurements*” (Quintile 4 educator).

### Quantitative logistics of implementation

Table 3 shows high learner penetration (97.2%), but lower learner exposure (48.0%) and parent exposure (54.4%). Many parents had signed to acknowledge reading through the workbook, however, the homework was not completed. Learner penetration was the highest at the quintile 4 school (99.1%) and the lowest at a quintile 1 school (92.3%). Learner exposure was the highest at the quintile 4 school (57.8%) and the lowest at a quintile 1 school (30.3%). Parent exposure was the highest at the quintile 2 school (62.3%) and the lowest at a quintile 1 school (35.5%).

**Table 3:** Penetration and exposure by lessons and by school.

		Quintile 1 Rural school 1	Quintile 1 Rural school 2	Quintile 2 Rural school	Quintile 3 Urban school	Quintile 4 Urban school	Total per school
		n (%)	n (%)	n (%)	n (%)	n (%)	%
**Lesson 1**	Learner penetration	116 (100.0)	99 (92.5)	78 (98.7)	140 (98.6)	92 (98.9)	**97.7**
**Obesity**	Learner exposure	69 (59.5)	59 (55.1)	72 (91.1)	99 (69.7)	67 (72.0)	**69.5**
	Parent exposure	70 (60.3)	48 (44.9)	70 (88.6)	96 (67.6)	68 (73.1)	**66.9**
**Lesson 2**	Learner penetration	115 (99.1)	100 (93.5)	79 (100.0)	139 (97.9)	92 (98.9)	**97.9**
**Nutrition**	Learner exposure	64 (55.2)	38 (35.5)	51 (64.6)	91 (64.1)	66 (71.0)	**58.1**
	Parent exposure	66 (56.9)	39 (36.4)	56 (70.9)	96 (67.6)	62 (66.7)	**59.7**
**Lesson 3**	Learner penetration	112 (96.6)	98 (91.6)	77 (97.5)	140 (98.6)	93 (100.0)	**96.9**
**Physical activity**	Learner exposure	53 (45.7)	28 (26.2)	44 (55.7)	68 (47.9)	61 (65.6)	**48.2**
	Parent exposure	61 (52.6)	35 (32.7)	44 (55.7)	89 (62.7)	60 (64.5)	**53.6**
**Lesson 4**	Learner penetration	113 (97.4)	98 (91.6)	78 (98.7)	138 (7.2)	91 (97.8)	**96.5**
**HIV/AIDS**	Learner exposure	24 (20.7)	13 (12.1)	28 (35.4)	48 (33.8)	27 (29.0)	**26.2**
	Parent exposure	57 (49.1)	38 (35.5)	41 (51.9)	82 (57.7)	49 (52.7)	**49.4**
**Lesson 5**	Learner penetration	114 (98.3)	99 (92.5)	77 (97.5)	137 (96.5)	93 (100.0)	**97.0**
**Communication**	Learner exposure	44 (37.9)	24 (22.4)	31 (39.2)	56 (39.4)	48 (51.6)	**38.1**
	Parent exposure	53 (45.7)	30 (28.0)	35 (44.3)	72 (50.7)	41 (44.1)	**42.6**
**Total**	Learner penetration	**98.3**	**92.3**	**98.5**	**97.8**	**99.1**	**97.2**
	Learner exposure	**43.8**	**30.3**	**57.2**	**51.0**	**57.8**	**48.0**
	Parent exposure	**52.9**	**35.5**	**62.3**	**61.3**	**60.2**	**54.4**

Table 4 shows the learner and parent exposure, by school and for the two activity tasks, i.e. the poster-making and the essay-writing activity. Learner exposure was the lowest at a quintile 1 schools (6.5%) and highest at a quintile 2 school (53.2%). Parent exposure was the lowest at a quintile 1 schools (35.5%) and highest at a quintile 2 school (70.9%). Some learners who submitted homework for the essay-writing activity wrote about their personal experiences of rape, abuse, and living with HIV. Others wrote stories of living without their parents who had died from HIV. The HIV theme provoked feelings of sadness rather than being a fun activity. Unfortunately, these issues could not be probed further as it was a story-writing task. Although there might not have been much truth behind the stories, the storylines were beyond the years of a pre-adolescent.

**Table 4:** Penetration and exposure by reinforcement activities and by school.

		Quintile 1 Rural school 1	Quintile 1 Rural school 2	Quintile 2 Rural school	Quintile 3 Urban school	Quintile 4 Urban school
		n (%)	n (%)	n (%)	n (%)	n (%)
**Poster-making activity**	Learner exposure	33 (28.4)	7 (6.5)	42 (53.2)	46 (32.4)	25 (26.9)
	Parent exposure	59 (50.9)	39 (36.4)	56 (70.9)	93 (65.5)	65 (69.9)
**Story-writing activity**	Learner exposure	24 (20.7)	13 (12.1)	28 (35.4)	48 (33.8)	27 (29.0)
	Parent exposure	57 (49.1)	38 (35.5)	41 (51.9)	82 (57.7)	49 (52.7)

There were notable differences in the characteristics of the poster submissions from school to school. None of the learners was provided with stationery. Learners from the quintile 4 urban schools submitted posters on expensive sheets of paper, used internet researched material and printed posters. Their pictures were drawn with coloured-pens or paints or were printed of the internet. The learners from other the other schools used low-cost lined paper torn from their books. They mostly used crayons and pictures cut from newspapers and magazines. All their information was copied directly from their textbook or the workbook. There was no evidence of additional research.

### Quality of implementation

Programme content was delivered with excellent facilitation skills, enthusiasm, preparedness, attitude, use of relevant examples, interaction style, and respectfulness, which resulted in positive learner participation. Three educators thought that most lessons produced a *“conducive and attentive atmosphere”*, and that *“learners were happy and engaged in the lesson”*. All educators understood and were knowledgeable about concepts being taught in every lesson. Educators stated that having the support of the researcher in the class, even if it were to observe, made them more confident. They answered learners’ questions and guided discussions in desired directions. However, when it came to discussions/questions around sexual-related content and behaviour, the educators looked to the researcher for assistance. Even though educators were trained on their health education syllabus and the intervention curricula, some educators showed a lack of confidence in teaching sexual-related content. When later probed, educators responded that discussing sex with young learners was uncomfortable.

Teacher-learner interactivity varied amongst implementers. One quintile 3 educator used a reading scenario strategy in her classes where learners were asked to read the notes from the workbook out loud to the class. The educator would then entertain a discussion around the subject. Learners’ reading was not fluent, and some struggled to pronounce the English words, but the educator used this strategy to reinforce reading skills, and improve English vocabulary. Another educator encouraged participation by directing her questions to learners who did not volunteer to answer questions. *“Children don’t answer questions in my class except for the ones that speak excellent English*. *Other children sit quietly and try not to answer because of their lack of confidence to speak English*, *so when I teach I ask questions to the quieter children”* (Quintile 1 educator).

Educators recommended adding teaching aids and charts to lessons as well as scenarios about how families live and actively exercise. Another educator said the combination of the theory and practical skills was useful to teach life skills. Some educators called for more in-depth notes while others said that the amount of notes in the workbook should be decreased.

Even after the training, five educators requested that the researcher be present to assist with the BMI measurements and pulse rate activities. First, some required the help of an additional person to complete the lesson in the given timeframe, and second, three said that they would feel more confident to carry out the activity under the supervision of the researcher, in case they experienced problems.

### Participant responsiveness

Aspects of participant responsiveness (the extent of which participants were engaged or involved in the activities and content of the programme) included their level of interest in the programme and perceptions about the relevance and usefulness of the programme.

Learners listened attentively and contributed to the class discussion at all but one school, where learners often tried to ‘bunk’ (truant) the lessons. At the start of each lesson, the educator would bring several boys back from the bathroom. Although she tried her best to keep their attention and discipline the class with a stick, it was difficult to control the learners. One lesson was rescheduled due to a memorial service for a child who had lost his life ‘train surfing’.

Educators thought that the ‘Broken Telephone Game’ designed to teach communication was *“enjoyable by all”*. A message was whispered from one child to the next to see how it changed over time. At the quintile 2 school, the message changed to include swear words.

*“Learners were eager to ask questions and share information about what they eat and buy from the tuck shop”* (Quintile 4 educator). This was not observed in the other schools, where learners were reluctant to share details about what they ate. Nonetheless, there were many ‘post-lesson’ questions from girls in the quintile 4 urban school, asked questions about obesity and healthy body image, such as, *“why am I overweight*, *when I play so much of sport*?*”*, and *“Why do my parents tease me about my weight*?*”* These girls seemed to be in the normal weight category, based on researcher observation rather than BMI calculations. This was also the only school in which learners requested that their weight measures not be read out loud.

In the lessons related to HIV, children in Quintiles 1 and 2 were eager to ask questions and share their home scenarios/stories. The learners in the quintile 4 school did not engage as fully in these lessons as they had done in those about obesity. *“learners were very interested in the lessons as they know of people who are affected directly by HIV”* (Quintile 1 educator). Although unnoticed by the researcher, an educator in a quintile 2 school said the HIV-related lessons created *“a tense atmosphere during class”*. However, during one of these lessons, *“learners opened up and shared their ideas and their experiences about HIV”* (Quintile 1 educator). One educator recommended adding more examples or case studies on the HIV topics.

The use of the workbook (extent to which a participant conducted activities specified in the materials) was initially high but decreased over time. The functionality of the workbook (the extent to which a participant continued to do any of the activities) was observed in two ways. First, even though all formal activities were not completed, most learners coloured in all the pictures and completed all the puzzles. Second, most learners were reluctant to hand in their books at the end of the programme, and many said that their books were stolen or lost. A possible reason for this was that they did not want to disclose that their parents had not participated. Parent participation through the workbook was conducive, with many parents offering positive comments and asking questions related to the topic.

In the quintile 1, 2 and 3 schools, stickers were offered as incentives, which encouraged and motivated the learners to do their work. They all looked forward to the stickers being be handed out. However, in the Quintile 4 school, *“Children hardly submit their work for stickers”* (Quintile 4 Educator). This was confirmed through observations.

The observing researcher attempted to play an insignificant role during lesson delivery and would only voluntarily participate, if asked directly by the educator to answer a question, which they were unsure of. It must be noted, however, that learners and educators were aware of the presence of the researcher during lesson delivery and would often look to gauge the researcher’s reactions to various occurrences. After the lesson, learners would often approach the researcher to have their individual questions related to the lesson delivered, answered privately.

### Appropriateness

All participating educators thought that the intervention was a fit for both rural and urban schools, all socio-economic groups, and learners from different ethnic and cultural backgrounds. *“These are the kids who need the information because some are facing the situation at home”* (Quintile 1 educator). Educators reported no language barriers during delivery of the programme. It was observed, however, that educators often reinforced instructions in the learners’ home languages. All content was also perceived to be age-appropriate for learners.

### Acceptability

The lessons and content met all the educators’ approval, and everyone welcomed the lessons into the Grade 6 life skills syllabus. *“The theory and skills were relevant and compatible with the school environment”* (Quintile 2 educator). “*The content was meaningful to the learners and to our communities*.*”* (Quintile 4 educator). All content was perceived to be acceptable to the learner age-group, to different socio-economic groups and to learners from different ethnic and cultural backgrounds. Although there was minimal sexual-related content to be taught, educators perceived that the sexual nature of some of the HIV-related content might be burdensome if learners asked difficult questions.

### Perceived sustainability

All educators felt that the programme could be sustained. First, however, educators could not guarantee that they would gain or retain the parents’ support if they tried to implement the programme on their own. *“When parents see an outsider doing something different*, *they might support the program*, *but on a normal day they don’t give us teachers the support”* (Quintile 1 educator). Another educator from the same school suggested that it would be difficult to maintain parent-level relationships or coalitions that were developed during the initial programme, without additional support. *“It would be simple to teach the child and let them relay the messages home to their parents but to get that parent to continue reading and signing for a longer period will be hard”* (Quintile 2 educator).

The second concern that educators voiced was if children would retain the acquired knowledge and be motivated to change behaviour once the intervention ended. *“When you are in front of the children*, *they remember you and what you are asking them to do but when you leave*, *they forget*. *I can teach them on HIV today*, *and when I ask them about it next year*, *they have forgotten the lesson”* (Quintile 1 educator). Another educator agreed that health messages needed to be continuously promoted and reinforced.

Third, they were uncertain if they would be able to teach the programme skills in their lessons or print the workbooks. All educators agreed that they would attempt to deliver the programme lessons of the original intervention, even after the programme ended, but that this would be difficult with the limited resources and equipment. *“The workbook and information in it*, *that was delivered*, *speak directly to the syllabus*. *I will use the workbooks again next year to make things easier to teach”* (Quintile 4 educator). Fourth, educators could also not guarantee that they would be teaching Grade 6 life skills in their schools the following year. Due to limited staff, subject educators often changed on a yearly basis. *“If time permits*, *I can use the existing resources and incorporate the additional sections into their lessons as enrichment activities*, *until such time as it can be adopted into the curriculum”* (Quintile 2 educator).

At the end of implementation, all educators felt that the programme should be included in the school curriculum and introduced into other schools. All educators concurred that the programme was beneficial, and two agreed to recommend that it be included into the school curriculum, to curriculum authorities during their school supervision visits. Programme diffusion and scale-up could be made possible through the provision of necessary organisational, political, community and financial support. According to educators, the following themes emerged, which could affect sustainability:

- funding stability (workbooks, equipment, training),
- political support (programme incorporated into curriculum, funding),
- organisational capacity (training, adequate resource allocation for programme),
- community support (parents/caregivers), and
- programme monitoring and evaluation (strategies to measure the differences in behaviour, attitudes, perceptions and health status).

## Discussion

*The CIrCLE of Life Initiative*, process evaluation findings indicate a high level of implementation fidelity, suggesting that the intervention was generally implemented and received as intended. The intervention was perceived by educators to be acceptable and appropriate to both learners and parents. However, findings show that implementer experiences with, and participant responsiveness to, HIV and obesity vary and some of the mechanisms that support implementation and how they function between the various health topics, schools, and contexts differ.

The exploration of context-outcome configuration is necessary to go beyond the generic ‘one recommendation fits all’ theory. Understanding the context of intervention delivery is critical to explain outcome as different interventions may have different causal processes and relationships with its context. Context differed between fee/non-fee paying schools, urban/rural schools, and the diseases, lifestyles, and quality of life experienced by learners in the different schools. Such facets of context need to be evaluated as it is relevant to understand implementation processes. There is no ‘one size fits all’ approach when it comes to implementation and, while programmes need to offer a degree of flexibility, there is a need to balance schools’ autonomy to adapt delivery with the contextual diversity of the different schools [42, 43]. For example, the results highlight that the quintile 4 urban school, which had a higher socio-economic status had better learner penetration and learner exposure, and relatively high parent exposure. The learners at this school were also eager to share about their nutrition and purchasing behaviour, unlike the lower socio-economic status schools that were part of the feeding scheme. Conversely, the learners in the quintile 4 school did not fully engage in certain lessons, such as HIV. This could be due to higher levels of baseline knowledge [44], which could suggest prior exposure to similar content. These learners engaged more in the obesity lessons. Such variations, by proxy of socio-economic status affect future implementation efforts within the contextual diversity of schools.

Recommendations were made to school management for certain schools to provide more supportive learning environments, not only for the implementation of the intervention but for routine school use. Supportive learning environments included smaller class sizes, clean and well-ventilated classrooms, essential teaching and learning resources, and an atmosphere conducive to concentration and learning. Educators are presented with more complex classrooms because of English as a first language, increasing numbers of learners, and with emotional and behavioural problems [45]. These issues create barriers for educators attempting to provide instruction and manage classroom behaviours among diverse learners [45]. Some of the key mechanisms and contexts identified in the initial implementation may also be important for long-term sustainability or scale-up. This study confirms the importance of such factors for a school-based programme and suggests their critical evaluation when developing/implementing school-based interventions.

Fidelity was a function of the implementers and encompassed both the quality and quantity of delivery [15, 46]. Assessing process skills and training methods were critical to improving the fidelity of the intervention [45]. Other important facets of implementation fidelity were educators’ knowledge levels, their interpersonal skills, and coverage of the specific content and processes used during delivery. The programme was flexible enough to allow for minor adaptations for the given contexts of delivery, as well as the skill level and degree of experience of the individual educators. Inter-school variation in delivery of the programme was observed. Educator delivery styles seemingly drove the evident variations in valence of adaptation. Although core teaching methods supported the educators to effectively deliver the programme, they also used their own experience to successfully tailor the programme to meet the needs of the learners, while preserving the essential functional components of the programme.

Regarding observations, the initial plan was for researchers to visit schools unannounced. However, this proved impractical due to the frequent changes in school schedules. Observation visits were therefore pre-arranged with schools, which meant schools had prior knowledge that the session would be observed, potentially influencing delivery. As the outcome evaluation was assessing effectiveness of the intervention in a ‘real world’ setting, the process evaluation was designed to avoid influencing its delivery. However, during observations, the researcher noted that some implementers perceived this as a source of support for intervention delivery. This posed a challenge on intervention delivery, and the researcher had to thus maintain a distance and not offer any specific feedback so as minimise influence.

The implementation of the intervention utilised a combination of delivery modes such as face-to-face delivery of lessons and the workbook. Educators perceived all modes to be acceptable to learners and their parents. The workbook was a means of indirectly involving parents. Not only was the workbook an acceptable mode, but it was also a feasible approach to involve parents. Slightly more than half of the parents were effectively reached in this manner.

Although the dose delivered was high, exposure rates of learners and parents were relatively low. Parent exposure was slightly higher than the learner exposure rate. Learner exposure was based on homework completed while parent exposure was based on signatures attained for reading through material. Homework/workbook activities were similar to traditional school homework and were least enjoyed by learners, which is evident from the low exposure rates. Not many learners completed the formal homework activities, yet most colouring-in activities and puzzles were completed by most. Using activities in the form of play may be a better way to encourage participation of learners [47, 48]. The degree of parental involvement in homework varied, but the workbook appeared to be an efficient way of reaching parents. Results relating to learner engagement with the intervention, or dose received, show that the frequency of learner engagement with the intervention was high during the study. Findings indicated that learner interest and engagement in activities decreased toward the end of the study.

As a transdisciplinary intervention, additional training and skills development for educators were necessitated. Even after adequate training, the results of the process evaluation provided an integrated understanding of the relationships between educator characteristics, and their training and experience. Educators’ attitudes and confidence influence the quality of implementation and the programme outcome [49]. Educators need to be better equipped to teach sexual-related content and to counsel and help learners deal with various problems. Professional development and training, therefore, become ever-more important to foster good health education practices in schools [43, 50]. Educators’ attitudes towards the programme and their levels of comfort with it are the most influential factors for quality of implementation. Educator pre-implementation training workshops should focus on strengthening their self-efficacy in communication [51] to implement any sexual-related content with high quality. Many learners and their families were affected by HIV. Understanding the plight of some of the participants may add context to how the disease has affected them and how the intervention could improve their health and well-being. Educators/implementers also require a level of sensitivity and skill-training in dealing with such issues [49].

The criterion for acceptability is personal [52]. Any two people can view the same programme and form different judgments. Individuals’ perceptions of acceptability, and their preferences and expectations, differ. The criterion for appropriateness, however, is technical or social [52]. A programme can be judged appropriate if it is seen as beneficial, or consistent with people’s norms or values. Two issues that influenced acceptability and appropriateness were language barriers and sexual content. South Africa has 11 official languages. With some exceptions, most public schools recently opted to teach using an English medium [53]. This affects the way learners comprehend, decipher and communicate lesson content. Language, as a perceived barrier, could influence acceptability and appropriateness of the intervention. Appropriateness and acceptability of communication about sexual-related topics is also viewed differently by certain cultures. It is a sensitive topic and not all parents find ease in its communication.

Implementation strategies have increased the development and translation of programmes into practice [54]; however, less attention is focused on post-implementation occurrences [55]. A sustainability evaluation determines how best to position a programme to ensure that it can, over time, sustain the salience of its core issues, such as its activities, organizational practices, and benefits to its recipients [56]. However, programmes typically need time to reach a certain level of maturity and allow health benefits to accrue to better understand what factors can promote long-term sustainability [57]. Sustainability may, therefore, only be observed well into, or even after, the implementation process. It is impossible to address all components of sustainability with any one method of data collection and analysis, as sustainability outcome evaluation requires exploration through longitudinal studies that measure multiple outcomes before, during and after programme implementation.

Taking cognizance of leading and lagging indicators of implementation may assist the sustainability process. While lagging indicators, e.g. sustainability, may reflect delays between when changes happen and when they can be observed, leading indicators, e.g. acceptability, can be useful as they signal future trends [27]. Sustainability, as a lagging indicator, was difficult to measure at this early stage after implementation, and the focus was, therefore, on conceptual development of the capacity for sustainability of the intervention. Sustainability capacity is the existence of structures and processes that allow a programme to leverage resources to effectively implement and maintain evidence-based policies and activities [57].

The findings included multiple stakeholder perspectives from those working on the ground and provided valuable insight into potential solutions for improving the sustainability of the programme to enable better support efforts [58]. The sustainability outcome evaluation recognised a set of organizational and contextual factors that could build capacity for maintaining the programme over time.

All educators unanimously concurred that the programme was beneficial, and should be included in the school curriculum and introduced into other schools. Programme diffusion and scale-up could be feasible through the provision of necessary community, organisational, and political support. Maintaining public health programmes that have shown benefit over time is a challenge due to the influence of human, informational, and financial resources [59, 60], as well as political and community support [57]. The sustainability of this intervention is very likely to be influenced by the broader policy environment. Not only should the programme be incorporated into the existing education curriculum, but it should also be supported by broader policy. Recently, two new South African policies have come into being, which could support sustainability of this programme. First, antiretroviral therapy are now available free-of-charge to all who are HIV positive; second the Health Promotion Levy on sugary beverages [61] has implications for food purchases.

### Strengths and limitations

The advantage of using several data collection methods allowed for a comprehensive analysis of intervention delivery in each school. Although the educator self-report logbooks created a risk of social desirability, the process was strengthened by the act of observation. The possible impact of direct observation and its influence on educators must also be acknowledged as it creates a more concerted effort to adhere to the protocol if educators know they are being observed. All logbooks were fully completed and the cross check of data between logbook and observation revealed high consistency. Qualitative questions in the logbook captured specific delivery methods used by educators, as well as their feedback about lessons, and was an added strength.

Learner-parent workbooks did not always correlate with independent evaluation. For example, it appeared as though learners sometimes signed on behalf of their parents. In these instances, the learner received the benefit of doubt, as educators confirmed that a vast majority of parents had low levels of education. Not having direct input such as open expressions of experiences and attitudes from learners and parents was seen as a limitation.

### Recommendations

- There is no ‘one size fits all’ approach when it comes to implementation and, while programmes need to offer a degree of flexibility, there is a need to balance schools’ autonomy to adapt delivery within the contextual diversity of schools.
- Educators need to be better equipped to teach sexual-related content and to counsel and help learners to deal with health problems. Professional development and training could foster good health education practices in schools.
- Using activities in the form of play may better encourage young learner participation.

## Conclusion

The comprehensive process evaluation of the implementation of *The CIrCLE of Life Initiative* was a valuable effort. It facilitated the evaluation of the implementation outcomes, described its processes, and acknowledged the fundamental characteristics that may contribute to the interventions’ success or failure. It has shown benefit to both research and practice in optimising future implementation efforts of the intervention.

## Supporting information

S1 File This file presents a Standards for Reporting Implementation Studies (StaRI) checklist with details regarding the implementation strategy that was used during the study. (DOCX)
